# Circular RNA hsa_circ_0076690 acts as a prognostic biomarker in osteoporosis and regulates osteogenic differentiation of hBMSCs via sponging miR-152

**DOI:** 10.18632/aging.103560

**Published:** 2020-07-27

**Authors:** Shijie Han, Mingjie Kuang, Chao Sun, Haifeng Wang, Dachuan Wang, Qian Liu

**Affiliations:** 1 Department of Orthopedics, The Provincial Hospital Affiliated to Shandong First Medical University, Jinan 250021, Shandong, PR China; 2 Department of Pain, Qilu Hospital of Shandong University, Jinan 250012, Sahndong, PR China

**Keywords:** osteoporosis, biomarker, hBMSCs, hsa_circ_0076690, miR-152

## Abstract

Objective: Osteoporosis is the most common skeletal disease world-wide. The aim of this study is to identify potential circRNA biomarkers for osteoporosis diagnosis and treatment, as well as their roles in regulating osteogenic differentiation.

Results: Hsa_circ_0076690 expression was significantly decreased in osteoporosis patients compared to control and showed an acceptable diagnostic value in clinical samples. Subsequently, hsa_circ_0076690 was identified to act as a sponge of miR-152. The expression of hsa_circ_0076690 was gradually increased during osteogenic differentiation while miR-152 showed a decreased expression trend. Moreover, osteogenic differentiation was promoted by hsa_circ_0076690 over-expression and remain unchanged by miR-152/hsa_circ_0076690 co-overexpression.

Conclusions: In conclusion, our study revealed that hsa_circ_0076690 may act as a potential diagnostic biomarker for osteoporosis patients and hsa_circ_0076690 could regulate osteogenic differentiation of hBMSCs via sponging miR-152.

Materials and methods: A total of 114 participants were enrolled in this study with ethics approvals. CircRNAs were identified by means of RNA-sequencing and qRT-PCR experiment. The clinical significance was measured by ROC curve analysis. Target relationship was validated by luciferase reporter assay. The osteogenic-associated biomarkers and ALP activity were detected by western blots.

## INTRODUCTION

Osteoporosis (OP) is a systemic skeletal disease with the main feature of reduction of bone density, low bone mass and increased risk of bone fracture [1–3]. The occurrence of osteoporosis was increased with age. According to the World Health Organization (WHO) criteria, osteoporosis is diagnosed as bone mineral density (BMD) below the average of the healthy (statistically as 2.5 or more standard deviations below the average) [4, 5]. Currently, the therapeutic approaches for osteoporosis are limited [6, 7]. Therefore, it is urgently needed to investigate the molecular genetics of osteoporosis to find novel therapeutic targets.

Circular RNAs (circRNAs) are a novel class of non-coding RNAs that characterized by a covalently closed continuous loop structure [8]. Numerous studies have shown that circRNAs are widely expressed in mammalian cells and act as potential regulators in cellular processes and disease pathogenesis [9]. The competing endogenous RNA (ceRNA) hypothesis indicates that circRNAs may competitively bind to microRNAs (miRNAs) to affect the expression of downstream genes [10, 11]. Human bone marrow mesenchymal stem cells (hBMSCs) are the source of bone-forming osteoblasts and play key roles in the renewal of osseous tissues [12–14]. Runt-related transcription factor 2 (RUNX2) was reported to be an important molecule during osteogenic differentiation and skeletal development [15, 16].

The present study characterized circRNAs expression profile in osteoporosis patient samples. Candidate circRNAs with abnormal expression were further validated by qRT-PCR experiment in 57 pairs of osteoporosis patients and non-osteoporosis healthy subjects. Subsequently, receiver operating characteristic (ROC) curve analysis was conducted to assess the diagnostic value of candidate circRNAs and found that hsa_circ_0076690 acted as a potential diagnostic biomarker for osteoporosis. Furthermore, the molecular mechanism of hsa_circ_0076690 was investigated in hBMSC cells and the results demonstrated that hsa_circ_0076690 might regulate osteogenic differentiation of hBMSCs through targeting miR-152.

## RESULTS

### CircRNA-seq analysis revealed that hsa_circ_0076690 and hsa_circ_0111433 were down-regulated in OP patients

**Figure 1:**
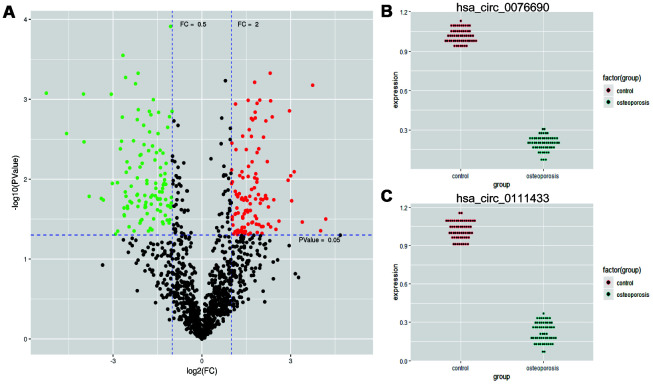
(**A**) Differentially expressed circRNAs were demonstrated by volcano plot (red: up-regulation green: down-regulation). (**B**, **C**) The down-regulation of hsa_circ_0076690 and hsa_circ_0111433 was validated by qRT-PCR in 57 pairs of OP and control samples.

The high quality clean reads were obtained from five pairs of OP and control samples by Illumina Hiseq 3000 and bioinformatics preprocessing. In total, 271 differentially expressed circRNAs were identified between OP and control samples, with |log_2_FC| ≥ 1 and *P*-value < 0.05 (Supplementary Table 2). The expression profiles of abnormally expressed circRNAs were demonstrated by volcano plot in Figure 1A. Among these circRNAs, we noticed that hsa_circ_0076690 and hsa_circ_0111433 were significantly down-regulated in OP patients compared to control (fold change: -20.77 and -16.29, respectively). To explore the role of hsa_circ_0076690 and hsa_circ_0111433 during osteoporosis pathogenesis, we validated the expression of two circRNAs in 57 pairs of OP and control samples by qRT-PCR assay. As shown in Figure 1B, hsa_circ_0076690 expression was significantly decreased in OP group (*P*-value < 0.01) compared to control. The expression level of hsa_circ_0111433 in OP showed a similar trend with hsa_circ_0076690 (*P*-value < 0.01, Figure 1C).

### Hsa_circ_0076690 acts as a potential diagnostic biomarker for OP

**Figure 2:**
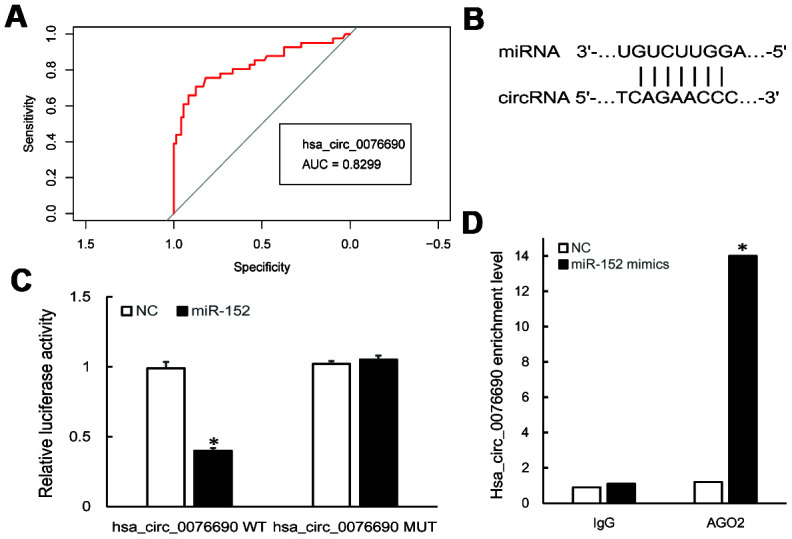
(**A**) ROC curve analysis of hsa_circ_0076690 in OP patients. (**B**) Predicted binding sites of hsa_circ_0076690 and miR-152. (**C**, **D**) Dual-luciferase reporter and RIP assay showed the direct binding of hsa_circ_0076690 and miR-152.

To examine the clinical significance of hsa_circ_0076690 and hsa_circ_0111433 for OP, the Pearson correlation analysis was conducted to measure the correlation between circRNAs expression and clinical parameters of OP patients. The statistical results showed that both the expression of hsa_circ_0076690 and hsa_circ_0111433 was significantly correlated with bone mineral density (BMD) and T-score (*P*-value < 0.05, r < -0.7, respectively), while had no significant correlation with age (r = -0.032 and 0.198, respectively) and Body Mass Index (BMI, r = 0.064 and 0.130, respectively) (Table 1). Subsequently, ROC analysis was conducted to investigate the accuracy of the two circRNAs serving as biomarkers for OP. As shown in Figure 2A, the AUC value was 0.8299 (*P*-value < 0.001) with 79% sensitivity and 85% specificity for hsa_circ_0076690, while hsa_circ_0111433 did not show an acceptable diagnostic value in clinical samples (data not shown).

**Table 1:** Pearson correlation analysis of circRNAs expression and clinical parameters of OP patients.

**Index**	**hsa_circ_0076690**	**hsa_circ_0111433**
Age	-0.032	0.198
BMI	0.064	0.130
BMD	-0.772*	-0.704*
T-score	-0.743*	-0.712*
CROSSL	0.101	0.128
TPINP	0.184	0.155
OSTEOC	0.177	0.126

*P-value < 0.05

### The potential regulatory axis of hsa_circ_0076690/miR-152/RUNX2

To investigate the molecular mechanism of hsa_circ_0076690 underlying osteoporosis pathogenesis, bioinformatics analyses was conducted. As predicted by miRanda and TargetScan software, hsa-miR-152-5p and hsa-miR-3678-3p were identified to have potential binding sites of hsa_circ_0076690 sequence (Figure 2B). Dual-luciferase reporter assay revealed that miR-152 could significantly inhibit the luciferase intensity in the hsa_circ_0076690 wild type compared to control and had no effect on hsa_circ_0076690 mutant (Figure 2C). Meanwhile, the luciferase intensity was not significantly reduced after co-transfection of miR-3678 and hsa_circ_0076690 wild type reporter (data not shown). Moreover, the RIP experiment indicated that hsa_circ_0076690 pulled down with anti-AGO2 was significantly enriched in miR-152 mimics group compared to control (*P*-value < 0.01, Figure 2D), indicating that hsa_circ_0076690 may function as a sponge for miR-152.

**Figure 3:**
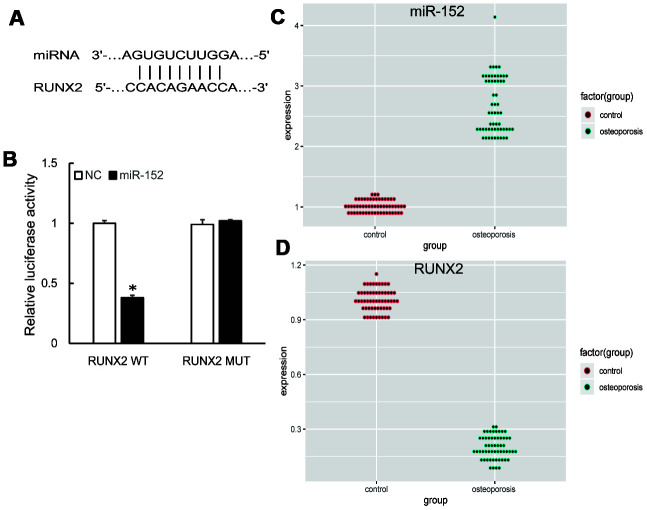
(**A**) Predicted binding sites of miR-152 and RUNX2. (**B**) Dual-luciferase reporter assay showed RUNX2 was a target of miR-152. (**C**, **D**) The up-regulation of miR-152 and down-regulation of RUNX2 were validated by qRT-PCR in 57 pairs of OP and control samples.

We further predicted the downstream targets of miR-152 by bioinformatics algorithm and found that RUNX2 was a potential target (Figure 3A). A subsequent dual-luciferase reporter assay revealed that the luciferase activity was repressed by miR-152 mimics in RUNX2 wild type, whereas the mutated luciferase reporter showed no changes (Figure 3B). In addition, qRT-PCR demonstrated that miR-152 expression was significantly increased in OP samples compared to control (Figure 3C), while RUNX2 expression was decreased (Figure 3D).

### Hsa_circ_0076690 regulated osteogenic differentiation of hBMSCs through targeting miR-152

**Figure 4:**
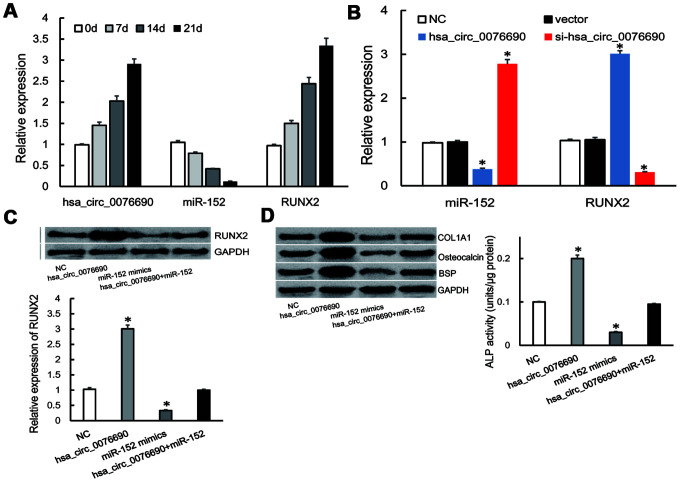
(**A**) The expression of hsa_circ_0076690, miR-152 and RUNX2 were measured by qRT-PCR during osteogenic differentiation of hBMSCs. (**B**) MiR-152 and RUNX2 expression levels were measured by qRT-PCR after transfected with hsa_circ_0076690 in hBMSCs. (**C**) RUNX2 expression was inhibited by miR-152 mimics and remained constant under miR-152/hsa_circ_0076690 co-overexpression. (**D**) The expression level of osteogenic-associated biomarkers and ALP activity were detected after transfected with hsa_circ_0076690, miR-152 mimics and miR-152/hsa_circ_0076690 co-overexpression in hBMSCs.

During the osteogenic differentiation of hBMSCs at 0, 7, 14 and 21 day, osteogenic-associated biomarkers were measured (Supplementary Figure 1A). We noticed that the expression of hsa_circ_0076690 and RUNX2 was accumulated (Figure 4A), on the contrary, miR-152 expression was gradually decreased in a time-dependent manner during osteogenic differentiation of hBMSCs (Figure 4A).

To further investigate the function of hsa_circ_0076690/miR-152/RUNX2 axis during osteogenic differentiation, qRT-PCR assays were performed in transfected hBMSCs (Supplementary Figure 1B). As a result, over-expression of hsa_circ_0076690 lead to the decreased expression of miR-152 and the increased expression of RUNX2 in hBMSCs, while si-hsa_circ_0076690 showed the opposite result (Figure 4B). In addition, the expression of RUNX2 was significantly inhibited by miR-152 mimics and remained constant under miR-152/hsa_circ_0076690 co-overexpression condition (Figure 4C). Moreover, the expression level of osteogenic-associated biomarkers and ALP activity were detected to measure the differentiation level of transfected hBMSCs. The results showed that osteogenesis differentiation was promoted by hsa_circ_0076690 over-expression and remain unchanged in miR-152/hsa_circ_0076690 co-overexpression condition (Figure 4D, Supplementary Figure 1C).

## DISCUSSION

With the development of RNA sequencing technology, a large number of circRNAs have been identified in mammalian cells [17]. Increasing evidence showed that circRNAs may act as novel candidate biomarkers for medical diagnosis on the disease [18, 19]. For example, the up-regulation of circRNA_002453 in lupus nephritis patients was associated with renal involvement [20]. Wu found that hsa_circ_0054633 was highly expressed in gestational diabetes mellitus patients and exhibited a significant diagnostic value [21]. By means of circRNA-seq and bioinformatics analysis, we identified hundreds of differentially expressed circRNAs between osteoporosis and control samples. Among these circRNAs, we found that hsa_circ_0076690 and hsa_circ_0111433 were significantly down-regulated in osteoporosis samples compared to control, with fold change of -20.77 and -16.29, respectively. The expression level of hsa_circ_0076690 and hsa_circ_0111433 was further validated by qRT-PCR experiments in 57 pairs of osteoporosis patients and non-osteoporosis healthy subjects. Subsequently, we performed Pearson Correlation and ROC curve analysis, the results revealed that hsa_circ_0076690 might serve as a candidate diagnostic biomarker for osteoporosis, which could distinguish patients with osteoporosis from healthy controls.

Recently, studies have focused on the potential regulatory role of circRNAs in biological events [9]. One hypothesis about its function is that endogenous circRNA may work as miRNA sponge by binding to miRNA and influence the biological function of miRNA [10, 11]. The molecular mechanism of hsa_circ_0076690 was further investigated in hBMSCs. By the means of bioinformatics and luciferase reporter assay, we found that hsa_circ_0076690 may function as a sponge for miR-152, which was reported to act as a prognostic marker for autoimmune disorders treatment and involved in various types of cancer [22]. A recent study showed that miR-152 could regulate glioma cell proliferation and apoptosis by targeting Runx2 [23]. Our dual-luciferase reporter assay revealed that miR-152 directly targeted RUNX2 in hBMSC cells and its expression was gradually decreased in a time-dependent manner during osteogenic differentiation of hBMSCs, while hsa_circ_0076690 showed an increasing trend. Moreover, the ALP activity and osteogenic-associated biomarkers were elevated by hsa_circ_0076690 over-expression and remain unchanged by miR-152/hsa_circ_0076690 co-overexpression.

In conclusion, our study revealed that hsa_circ_0076690 may act as a potential diagnostic biomarker in osteoporosis patients. Moreover, hsa_circ_0076690 could regulate osteogenic differentiation of hBMSCs through targeting miR-152.

## MATERIALS AND METHODS

### Study subjects and ethics statement

In total, 57 pairs of osteoporosis patients and non-osteoporosis healthy subjects were enrolled in this study. None of the subjects had metabolic diseases that affect bone metabolism or received medical treatment before. Clinical parameters were recorded from all the participants, including age, body mass index (BMI), BMD, T-score, β-crosslaps (β-CROSSL), N-terminal osteocalcin (OSTEOC) and Total Procollagen Type1 Intact N-terminal Propeptide (TPINP), etc (Supplementary Table 1). This study was approved by Ethics Committee of Qilu Hospital of Shandong University and was in accordance with Helsinki Declaration. The written informed consents were signed by all participants.

### CircRNA-seq analysis

Five pairs of serum or plasma samples from osteoporosis patients and healthy controls were subjected to circRNA sequencing using Illumina Hiseq 3000 (Illumina, CA, USA). Firstly, total RNAs were extracted from samples using (TRIzol CA, USA) following the instruction. After measuring the quantity and quality of RNAs, Rnase R was used to remove linear transcripts. Subsequently, cDNA library was built for circRNA sequencing following the instruction of TruSeq RNA Sample Prep Kit (Illumina, CA, USA). FastQC and cutadapt software were used to obtain high quality clean reads for further analysis. GRCh38 genome and circBase were used for circRNAs annotation. EdgeR package was used to filter the differentially expressed circRNAs between osteoporosis and control group (log_2_FC ≥ 1 and *P*-value < 0.01).

### Bioinformatics and statistical analysis

Differentially expressed circRNAs between osteoporosis and control group was demonstrated by Volcano plot, which was drawn by ggplot2 package. The diagnostic values of candidate circRNAs were measured by ROC curve analysis and the areas under the curve (AUC) were calculated by R. Target prediction was performed by TargetScan and miRanda software. All experiments data are representative of independent triplicate experiments and the statistical analysis was performed using R. Statistically significant was set to *P*-value < 0.05.

### Osteogenic differentiation of hBMSCs

The hBMSC cells were purchased from Ribio company (Beijing, China) cultured in culture medium supplemented with 12% fetal bovine serum (24-well plates, 37 °C). The hBMSCs induction process was conducted by supplying the cells with osteogenic differentiation medium and changed every three days. HBMSC cells were cultured for twenty-one days and the osteogenic associated biomarkers including ALP, Osteocalcin, β-catenin, BMP2 and RUNX2 were detected to measure the osteogenic differentiation process.

### Cell transfection and ALP measurement

HBMSC cells were cultured in six-well plates and transfected with Lipofectamine 3000 for 6h according to the manufacturer’s instruction. The negative controls (NC), miR-152 mimics and si-hsa_circ_0076690 were purchased from Genechem company (Shanghai, China). The ALP activity was measured after transfected for 14 days using a commercial kit according to the instruction. Absorption was measured at 405 nm.

### Quantitative real time-polymerase chain reaction (qRT-PCR)

Total RNAs were extracted from osteoporosis samples, control samples and transfected hBMSCs using TRIzol reagent (Invitrogen, CA, USA) according to the protocol. The reverse-transcription reaction was conducted using Reverse Transcriptase kit (Invitrogen, CA, USA). The Real-time PCR was performed to detect the expression of candidate circRNAs in tissue samples and specific molecule in transfected hBMSC cells. A SYBR Green PCR kit (Takara, Japan) was used following the instrument: denaturation for 180 s at 93 °C and followed by 30 cycles at a Real-Time PCR Applied Biosystems. The relative expression level was determined by 2^-ΔΔCt^ method, GAPDH and U6 were used as references.

### Western blot and Alizarin red staining (ARS)

The hBMSC cells from different groups were lysed with protein-extraction reagent (Roche, Mannheim, Germany) following the instrument. The BCA Protein Assay Kit (Beyotime, China) was used to measure protein concentration. Then proteins were separated by 12% sodium dodecyl sulfate-polyacrylamide gel electrophoresis (SDS-PAGE) and transferred to PVDF membrane to incubate with primary antibody. Finally, the membrane was washed (three times) and further incubated with secondary antibody. The bands were visualized by ECL (Millipore) following the instrument. ARS staining was performed at 14d and 21d during osteogenic differentiation by treating cells with 0.1% ARS-Tris-Hcl (1h, 37°C), then the mixture was washed by distilled water and ARS level was examined by microscope.

### Dual-luciferase reporter assay and RNA immune-precipitation

The potential binding sequences between RNA molecules were synthesized and cloned into pGL3-Basic luciferase vector. Luciferase plasmid and miR-152 NC/mimics were transiently transfected into cells with Lipofectamine 3000 for 24h following the protocol. The luciferase activities were then measured using an applied system (Promega, USA). The RNA immunoprecipitation (RIP) experiment was conducted in transfected cells using an RNA Binding Protein Kit (Millipore, USA) and human anti-Ago2 antibody following the protocols. IgG was used as negative control and binding output was measured by qRT-PCR.

## Supplementary Material

Supplementary Figure 1

Supplementary Tables

Supplementary Table 2
