# Supplementary material for: Circular RNA hsa_circ_0076690 acts as a prognostic biomarker in osteoporosis and regulates osteogenic differentiation of hBMSCs via sponging miR-152
Source: Aging (Albany NY). 2020 Jul 27;12(14):15011–20. doi: 10.18632/aging.103560 (PMC7425508; doi:10.18632/aging.103560)
Supplement: Supplementary Figure 1 [file aging-12-103560-s003..pdf]

SUPPLEMENTARY FIGURE

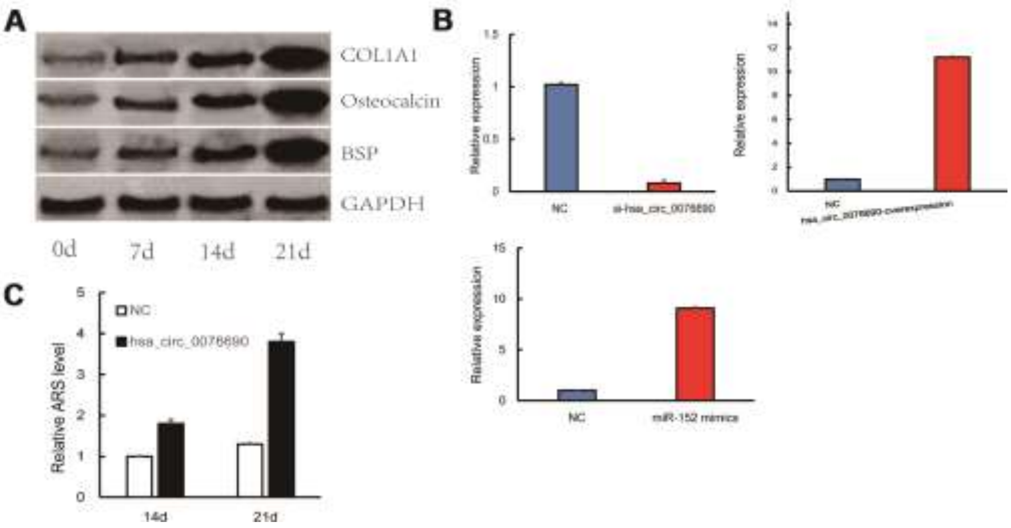

Supplementary Figure 1. (**A**) The differentiation level of hBMSCs were measured. (**B**) HBMSC cells were transfected with different conditions. (**C**) ARS level that affected by circ_0076690 during osteogenic differentiation.
