# Supplementary material for: Circular RNA hsa_circ_0076690 acts as a prognostic biomarker in osteoporosis and regulates osteogenic differentiation of hBMSCs via sponging miR-152
Source: Aging (Albany NY). 2020 Jul 27;12(14):15011–20. doi: 10.18632/aging.103560 (PMC7425508; doi:10.18632/aging.103560)
Supplement: Supplementary Tables [file aging-12-103560-s002..pdf]

SUPPLEMENTARY TABLE

Supplementary Table1. Characteristics of osteoporosis patients and healthy control.

Clinical index (mean ± SD)	osteoporosis	control
Age	62 ± 6.00	61 ± 7.00
BMI	20.1 ± 3.96	21.3 ± 3.44
BMD (g/cm2)	0.58 ± 0.10	0.79 ± 0.05
T-Score Lumbar Spine (L2-L4)	-2.7 ± 0.19	0.4 ± 0.93
CROSSL (ng/mL)	0.77 ± 0.13	0.50 ± 0.07
TPINP (ng/mL)	63.8 ± 7.01	51.2 ± 6.86
OSTEOC (ng/mL)	22.9 ± 2.6	14.17 ± 2.5
